# Reaching the Unreached: Providing Quality Care to HIV-Infected Children through Telemedicine—An Innovative Pilot Initiative from Maharashtra, India

**DOI:** 10.1155/2020/6432476

**Published:** 2020-10-23

**Authors:** Mamta Manglani, Yashwant Gabhale, Mamatha Murad Lala, Sudha Balakrishnan, Khanindra Bhuyan, B. B. Rewari, Maninder Singh Setia

**Affiliations:** ^1^Pediatric Centre of Excellence, Department of Pediatrics, LTM Medical College and General Hospital, Mumbai, India; ^2^UNICEF, India; ^3^HIV/STI/HEP WHO, Regional Office for South East Asia, (Formerly, National Program Office, CST, National AIDS Control Organization, India), India; ^4^MGM Institute of Health Sciences, Navi Mumbai, Maharashtra 410209, India

## Abstract

**Background:**

The National AIDS Control Organization (NACO) of India created the Regional Pediatric Antiretroviral Therapy (ART) Center; this was subsequently upgraded to seven Pediatric Centers of Excellence (PCoEs) to strengthen the quality of treatment and care of children living with HIV/AIDS (CLHAs). In October 2013, the pediatric HIV telemedicine initiative, an e-decentralized (care provided by local healthcare providers and support provided by a central agency through telemedicine facilities) model of expert pediatric HIV care and referral services, was established as a pilot project at the Pediatric Center of Excellence for HIV Care in Maharashtra. We designed the present study to compare management, compliance to ART, and mortality in children in the ART centers linked to the PCoE through telemedicine versus those that are not linked to the PCoE.

**Methods:**

It was a retrospective cross-sectional study of secondary data from CLHAs from October 2013 through August 2015 in the ART centers to document the intermediate outcomes and to determine if the initiative has improved the quality of care for the CLHAs enrolled in the linked ART centers with nonlinked ART centers. The centers in which the telemedicine sessions were conducted regularly were called linked-regular centers and in whom it was conducted irregularly (less than the median of 12 videoconference cases), it was called a linked-irregular center. Data from 2803 children in 31 linked (1365 in irregular and 1438 in regular centers) and 2608 children in 28 nonlinked centers were analyzed. The outcomes in children in the pre-ART group (ART naïve) were (1) alive on pre-ART, (2) lost to follow-up on pre-ART, (3) death during the pre-ART period, (4) eligible but not initiated on ART, and (5) missing baseline and latest CD4 counts. The outcomes of children on ART were (1) alive on ART, (2) lost to follow-up on ART, (3) death on ART, and (4) missing baseline and latest CD4 counts.

**Results:**

We found that a higher proportion of children in the linked-regular centers (79% vs. 70%, *p* < 0.001) and linked-irregular centers (76% vs. 70%, *p* = 0.04) was alive compared with that in the nonlinked centers in the pre-ART group. In this group, the proportion of children with missing baseline CD4 counts and latest CD4 counts was significantly low in linked (regular centers) centers. In the ART group, we found that a higher proportion of children in the linked-regular centers was alive compared with that in the linked-irregular centers (77% vs. 69%, *p* < 0.001); the proportion was not significantly different in nonlinked centers (77% vs. 78%, *p* = 0.56). In this group, the proportion of missing baseline CD4 counts was significantly lower in the linked-regular centers (3% vs 13%, p<0.001) and linked-irregular centers (1% vs. 13%, *p* < 0.001) compared with that in the nonlinked centers. Furthermore, the latest CD4 counts were missing in a significantly lower proportion of children in the linked-regular centers compared with those in the linked-irregular centers (6% vs. 18%, *p* < 0.001) and nonlinked centers (6% vs. 18%, *p* < 0.001).

**Conclusion:**

Our study shows that the centers linked through telemedicine performed better in terms of patient care and treatment, with a lesser loss to follow-up and lesser deaths in CLHA. Overall, this pilot project of telemedicine for pediatric HIV has been proven to be acceptable, feasible, and effective in improving the quality of care for children living with HIV across the state of Maharashtra.

## 1. Introduction

The Indian National AIDS Control Program (NACP) is globally acclaimed as one of the most successful programs, attributable to its successful prevention-focused policies, evidence-driven strategies, and openness for innovations. With the estimated number of new HIV infections in 2017 being 87580, a drastic decline of nearly 85% has been achieved since 1995 [1]. There has been a steady increase in the number of Antiretroviral Therapy Centers (ARTCs) providing care to HIV-infected individuals, with a total of 537 ART centers spread across the country; since the launch of the pediatric ART initiative in November 2006 by the National AIDS Control Organization (NACO), over 80000 children living with HIV (CLHA) are in active care [1].

Although the recommended pediatric ART formulations are available at the ART centers, most of these centers are not managed by pediatricians or specialists in pediatric HIV. Hence, NACO created the Regional Pediatric ART Centers (seven across the nation based on the expertise and data related to pediatric HIV), which were referral centers for the various ARTCs. These were subsequently upgraded to seven Pediatric Centers of Excellence (PCoEs) to strengthen the quality of treatment and care of HIV-infected children. The seven PCoEs in the country are as follows: (1) Lokmanya Tilak Municipal Medical College and General Hospital, Mumbai, Maharashtra; (2) Institute of Child Health, Chennai, Tamil Nadu; (3) Government Medical College and Hospital, Kolkatta, West Bengal; (4) Kalawati Saran Children's Hospital, New Delhi; (5) J N Hospital, Imphal, Manipur; (6) Indira Gandhi Children's Hospital, Bengaluru, Karnataka; and (7) Niloufer Hospital, Hyderabad, Telangana. These PCoEs are responsible for the care and treatment of children, management of complicated opportunistic infections, specialized laboratory services, training, and research-related activities. Furthermore, these centers also provide specific counseling services on nutrition, age-appropriate disclosure, and any other specific counseling needs [1].

Complicated pediatric HIV cases that cannot be managed at the peripheral centers get referred to the closest higher center or the PCoE for expert pediatric HIV care, but many of these cases fail to report to the referral center due to financial and time constraints, leading to further deterioration or death in these children. However, there are a number of challenges in providing pediatric HIV treatment and quality care to date, mainly due to the nonavailability of expertise at the peripheral centers and the staggering distances between these peripheral centers and the center of excellence for referral services.

To address this issue, a joint field visit of experts for needs assessment from PCoE along with the Maharashtra State AIDS Control Society (MSACS) and United Nations Children's Fund (UNICEF) was undertaken to a few peripheral ART centers (Nashik and Malegaon) in September 2013, to explore the option of telemedicine in providing mentoring support to all ARTCs in the state. In the visit, it was observed that while the ART Medical Officers (MOs) were committed to provide care and treatment to the CLHA, there was a need to enhance their capacities in managing pediatric HIV cases. This was strikingly seen in the ARTCs where pediatricians were not the ART Medical Officers (MOs). There were 52 ART centers out of 60, where ART MOs were not pediatricians, thereby justifying the need to strengthen the capacities. With this background, a unique telemedicine initiative was planned wherein the National Health Mission, Government of Maharashtra would provide the software as well as the link with all the centers that were in proximity to the ART centers, MSACS would provide the logistic support, UNICEF would provide the equipment and technical and project management support, and the PCoE would offer the expertise and mentoring support and build the capacities of the service providers at the linked-ART centers through telemedicine. The plan of this e-decentralized approach in Maharashtra clearly indicated that the use of telemedicine seemed feasible, acceptable, and viable and had the potential to improve care for HIV-infected children, leading to improved outcomes in resource-poor settings, besides resulting in cost savings, both at the provider and the client end.

Thus, in October 2013, the pediatric HIV telemedicine initiative, an e-decentralized model (wherein the peripheral ARTCs would receive virtual hand-holding and mentoring regularly through telemedicine for capacity building that translates into management across all patients enrolled in their centers locally, without them having to physically refer their patients to tertiary centers often) of expert pediatric HIV care and referral services, was established as a pilot project at the Pediatric Center of Excellence for HIV Care in Maharashtra, functioning out of Lokmanya Tilak Municipal Medical College and General Hospital, Sion, Mumbai, in a multipartner collaboration with NACO, MSACS, National Health Mission in Maharashtra (NHM), Municipal Corporation of Greater Mumbai (MCGM), and UNICEF. The vast state of Maharashtra with many difficult-to-reach tribal and hilly areas has a total of 86 ARTCs, providing HIV treatment and care services to a huge number of HIV-infected patients (adult HIV prevalence 0.42%, PLHIVs: 315849, CLHIVs: 26807, and children on ART: 13913) [2]. Linkage of 35 of these 86 ARTCs in the state was established through “The Pediatric HIV Telemedicine Initiative” project for delivery of virtual expert services, designed to reach the unreached HIV-infected infants, children, and adolescents with quality care. The expert services included real-time consultation with the patients and their caretakers along with the team of doctors and counselors providing them care at the peripheral level done on a planned as well as need basis. On a planned basis, two centers were covered every day during which the peripheral ART teams discussed at least 3 of their randomly selected patients for an expert opinion. Follow-up of these patients was provided during their next round of “planned videoconference” (scheduled by rotation with all linked centers which automatically happened 4-6 weeks later) or earlier if warranted. On a need basis, any linked center could request for a “need-based videoconference” if they needed urgent expert opinion for a walk-in patient, out of their schedule. The purpose was to facilitate easy access to expert guidance on various aspects of HIV management such as HIV diagnosis, ART initiation and follow-up, management and/or prophylaxis of opportunistic infections, drug toxicities, treatment failure, nutrition and adherence counseling, and age-appropriate disclosure. In addition, mortality reviews were conducted; mentoring and training sessions were held regularly, thereby building the overall capacity of the healthcare providers [3]. Through this initiative, currently, about 50% of ART centers in 36 districts of Maharashtra (excluding Mumbai) are linked to PCoE through telemedicine.

With this background, the present study was designed to compare the outcomes in children in the ART centers linked to the PCoE through telemedicine versus those that are not linked to the PCoE. These outcomes were management of HIV-infected children, compliance to ART, and mortality.

## 2. Methods

### 2.1. Study Design

It was a retrospective cross-sectional analysis of secondary data to determine if there is a difference in the quality of care for the CLHAs enrolled in the linked ART centers with nonlinked ART centers. Data from October 2013 through August 2015 were included for the present analysis (indicated in Table 1 showing the sample description).

### 2.2. Data Source

All children up to 18 years from 31 linked ARTCs (that had completed at least five “planned videoconference” sessions till August 2015 (these were the minimum number of videoconference sessions that were planned, scheduled, and conducted across all the centers in Maharashtra)) and 28 nonlinked centers were included in the present study.

### 2.3. Study Population and Sample Selection

Of 31 linked centers, 12 were classified as irregular centers (less than median number [4] of videoconference sessions conducted across the centers), and 19 centers were classified as regular centers (more than or equal to the median number of regular VCs).

### 2.4. Study Variables and Outcomes

The variables in the datasets were (1) district of the ART site, (2) age of the individual, (3) sex of the individual, (4) pre-ART status, (5) ART status, (6) baseline CD4 counts, and (7) latest CD4 counts. Besides these individual-level variables, we created a file of contextual-level variables. These variables were (1) nature of the ART center (municipal corporation hospital, civil hospital, medical college, and subdistrict hospital), (2) number of planned videoconferences conducted (the value was zero for nonlinked centers), and (3) presence/absence of pediatrician in the ART center.

We used the pre-ART (ART naïve) and ART (on ART) status for our analysis. In the pre-ART children, we evaluated the following outcomes: (1) alive on pre-ART, (2) lost to follow-up on pre-ART, and (3) death during the pre-ART period. In CLHAs who were on ART, we evaluated the following outcomes: (1) alive on ART, (2) lost to follow-up on ART, and (3) death on ART.

Besides these, additional outcomes were (1) eligible but not initiated on ART, (2) baseline CD4 counts, (3) baseline CD4 count missing, (4) latest CD4 count, and (5) latest CD4 count missing. The last available data point was used for outcome analyses in the pre-ART and ART groups.

### 2.5. Statistical Analysis

We calculated the means and standard deviations for continuous variables and proportions for categorical variables. The means were compared using the *t*-test (for two groups) and the proportions were compared using the chi-square test or Fisher's exact test for low expected cell counts.

We then used random-effects multilevel logistic regression models to study the association between the explanatory variables and the outcomes across these centers. The primary explanatory variable was the type of center: (1) nonlinked centers (28), (2) linked center with less than the median number of regular videoconferences (12), and (3) linked centers with more than or equal to the median number of regular videoconferences (19).

The two levels in our analyses were the individual child (level 1) and the center (level 2). We built the models in the following sequences: (1) null models without any explanatory variables (to assess the level 2 variance), (2) models with only one explanatory variable, and (3) multivariate models with all the individual- and contextual-level variables. The following variables were included in the multivariate models: linked/nonlinked center (primary explanatory variable), age, gender of the child, CD4 counts at baseline, nature of the healthcare center, and presence of a pediatrician in the center. We used the Akaike Information Criteria (AIC) to assess the fit of various models.

The study was approved by the Ethics Committee of Lokmanya Tilak Municipal Medical College and General Hospital.

## 3. Results

A total of 2803 children in 31 linked (1365 in irregular and 1438 in regular centers) and 2608 children in 28 nonlinked centers were analyzed (Table 2).

The majority of the children in these centers were in the age group of 10-14.9 years (32%); the age distribution did not differ significantly across these three types of centers (*p* = 0.49). However, the linked centers (particularly linked-irregular centers) had a significantly higher proportion of males compared with females (*p* = 0.009) (Table 2). We found that a higher proportion of medical college and municipal corporation hospitals was not linked to telemedicine. However, a significantly higher proportion of civil hospitals and subdistrict hospitals was linked by telemedicine facilities (*p* < 0.001). The median number (range) of medical officers in these centers was 2 (1-3); it did not differ significantly across types of centers. The majority of the medical officers in these centers had a postgraduate degree (82%); however, only 27% of the centers had a pediatrician. The median (range) experience of these medical officers in HIV care was 4 (0.6–10) years; the median experience did not differ across types of centers (Table 2).

**Figure 1:**
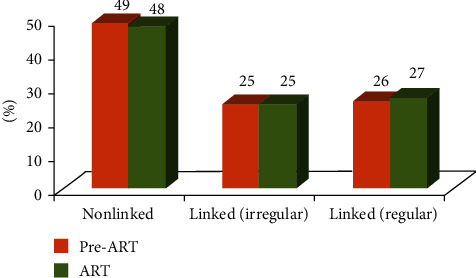
The percentage of children in pre-ART and ART groups, by type of ART center in Maharashtra.

Of the total 5411 children, 3676 (68%) were on ART and 1735 (32%) were in the pre-ART group. The proportion of children in the “pre-ART group” and “on ART” did not differ significantly across the three types of centers (Figure 1). The baseline CD4 counts were missing in 4% of children in the linked-regular and nonlinked centers and in 6% of children in the linked-irregular centers. However, the latest CD4 counts were missing in only 9% of children in the linked-regular centers compared with 25% in the linked irregular centers and 21% in the nonlinked centers (*p* < 0.001).

We have presented additional data on these parameters in Table 2.

### 3.1. Pre-ART Children

We found that a higher proportion of children in the linked-regular centers (79% vs. 70%, *p* < 0.001) and linked-irregular centers (76% vs. 70%, *p* = 0.04) was alive compared with that in the nonlinked centers. However, there was no significant difference between linked-regular and linked-irregular centers (79% vs. 76%, *p* = 0.21). Reported mortality was similar across the three types of centers (4%). Lost to follow-up was lower in linked centers (regular and irregular) compared with that in nonlinked centers (6% vs. 5% vs. 8%, *p* = 0.09) (figures are presented in respective sequence); the difference was not statistically significant. We found that a significantly lower proportion of children in the linked-irregular centers were not started on ART in spite of being eligible for initiating it (as per the existing national guidelines); the proportion was similar in linked-regular and nonlinked centers (4% vs. 1% vs. 4%, *p* = 0.003) (figures are presented in respective sequence) (Table 3).

Among centers that had a medical officer with a postgraduate degree (*p* < 0.001) or a pediatrician (*p* = 0.03), a higher proportion of children was alive in the linked-regular centers compared with that in the linked-irregular centers or nonlinked centers (Table 4).

The proportion of children with missing baseline CD4 counts (6% vs. 8% vs. 20%, *p* = 0.001) and latest CD4 counts (13% vs. 30% vs. 34%, *p* < 0.001) was significantly lower in the linked-regular centers compared with that in the nonlinked and linked-irregular ART centers of civil hospitals (figures are presented in respective sequence). Similar results were found in centers that did not have a pediatrician. Furthermore, even in centers with a pediatrician, the latest CD4 counts were missing in a significantly lower proportion of children in the linked regular centers compared with linked-irregular (7% vs. 45%, *p* < 0.001) and nonlinked centers (7% vs. 34%, *p* < 0.001) (Table 3).

We have presented additional details about these outcomes in the pre-ART children in Tables 3 and 4.

### 3.2. Children on ART

We found that a higher proportion of children in the linked-regular centers was alive compared with that in the linked-irregular centers (77% vs. 69%, *p* < 0.001); the proportion was not significantly different in nonlinked centers (77% vs. 78%, *p* = 0.56). Reported mortality was higher in the linked-regular centers compared with that in the linked-irregular and nonlinked centers (6% vs. 4% vs. 4%, *p* = 0.02) (figures are presented in respective sequence). The proportion of children who were lost to follow-up was lower in linked-regular centers and nonlinked centers compared with that in linked-irregular centers (4% vs. 9% vs. 4%, *p* < 0.001) (figures are presented in respective sequence) (Table 5).

Among centers based in civil hospitals, a lower proportion of children was “lost to follow-up” in the linked-regular centers compared with that in the linked-irregular centers (4% vs. 8%, *p* = 0.009) or nonlinked centers (4% vs. 9%, *p* = 0.04). We found the baseline CD4 counts were missing in a significantly lower proportion of children in the linked-regular centers (3% vs. 13%, *p* < 0.001) and linked-irregular centers (1% vs. 13%, *p* < 0.001) compared with nonlinked centers. Furthermore, the latest CD4 counts were missing in a significantly lower proportion of children in the linked-regular centers compared with linked-irregular centers (6% vs. 18%, *p* < 0.001) and nonlinked centers (6% vs. 18%, *p* < 0.001).

Linked-regular centers performed better compared with linked irregular or nonlinked centers as far as latest CD4 counts were concerned irrespective of the presence of a postgraduate medical officer or a pediatrician (Table 6).

We have presented additional details about the outcomes and quality of care indicators in these children in Tables 5 and 6.

### 3.3. Multivariate Models

In the pre-ART group, we found that children who were in the age group of 5-9.9 years (OR: 2.3, 95% CI: 1.5, 3.5), 10-14.9 years (OR: 2.1, 95% CI: 1.4, 3.2), and 15-18 years (OR: 1.9, 95% CI: 1.2, 3.1) were significantly more likely to be alive compared with children who were in the age group of 0-4.9 years. We also found that children with baseline CD4 counts in the range of 201-350 (OR: 1.9, 95%: 1.0, 3.7), range of 351-500 (OR: 12.6, 95% CI: 7.4, 21.6), or those greater than 500 (OR: 15.6, 95% CI: 9.5, 25.7) were more likely to be alive compared with those with baseline CD4 counts of less than 200.

In the ART group, we found that children who were in the age group of 5-9.9 years (OR: 1.3, 95% CI: 1.0, 1.8), 10-14.9 years (OR: 1.8, 95% CI: 1.4, 2.4), and 15-18 year (OR: 1.9, 95% CI: 1.4, 2.5) were significantly more likely to be alive compared with children who were in the age group of 0-4.9 years. We also found that children with baseline CD4 counts in the range of 201-350 (OR: 1.3, 95%: 1.0, 1.6) and those greater than 500 (OR: 2.0, 95% CI: 1.6, 2.6) were more likely to be alive compared with those with baseline CD4 counts of less than 200.

We did not find any significant associations between any demographic variable or nature of the centers and lost-to-follow-up data in children in the pre-ART and ART groups. Each outcome was assessed in the multivariate models separately for the pre-ART and ART groups.

## 4. Discussion

The data for 5411 children who had visited 31 linked and 28 nonlinked centers during the study period (between October 2013 and August 2015), when analyzed, revealed that the majority of the children across all three types of centers (linked centers, linked-irregular centers, and nonlinked centers) fell into the age group of 10-14.9 years. A higher proportion of children in the linked-regular centers was alive compared with that in the linked-irregular centers and nonlinked centers which was statistically very significant; the proportion of children who were lost to follow-up was lower in linked centers. Linked-regular centers performed better compared with linked-irregular or nonlinked centers as far as latest CD4 counts were concerned irrespective of the presence of a postgraduate medical officer or a pediatrician. As per the multivariate model analysis, in both the pre-ART and ART groups, more children were alive and in active care in the linked centers compared to linked-irregular and nonlinked centers; children less than 5 years of age and children with a baseline CD4 less than 200 cells/c.mm were more likely to have died in both the groups as expected.

The centers that had medical officers with a postgraduate degree (not necessarily in pediatrics) had a higher proportion of children alive on ART, and their CD4 monitoring (both baseline and latest) was more likely to be done in the linked-regular centers compared with linked-irregular centers or nonlinked centers; it was also noted that the children in care in medical colleges were significantly less likely to have died compared with those in the civil hospitals.

The linkage to PCoE through telemedicine also perhaps contributed the linked-ART centers to avert an annual average 2.2 loss of patients to follow-up per ART center (*p* = 0.04) among CLHA as compared with their nonlinked counterparts over this time period.

As seen in our study, a higher proportion of medical college and municipal corporation hospitals were not linked to telemedicine. Most of these centers have senior faculty and/or subject area expertise. Thus, in the initial phase of telemedicine connection, these centers were not linked to the PCOE. Other hospitals such as civil and subdistrict hospitals may not often have such expertise. Thus, they were linked to PCOE and became linked centers. As observed in our data, linked-regular centers were best on most of the indicators. The nonlinked centers often performed better in some indicators compared with linked-irregular centers. It is quite likely that the presence of subject area experts helped improve the outcomes, though it may be lower than linked-regular centers. It is important, however, to note that linked-irregular centers performed poorer on some indicators compared with nonlinked centers. Thus, potential lack of subject area expertise and irregular videoconferences with PCOE may have contributed to poor indicators, whereas regular videoconference sessions with PCOE helped improve the outcomes in these centers.

Several reviews, evaluating different types of TM technologies and telephone consultations, have been published. It has been highlighted that telemedicine is an important mechanism for service delivery in healthcare settings, both in resource-rich and resource-poor settings [5–11]. For HIV-infected patients, such service deliveries have been shown to be associated with virologic suppression and higher CD4 counts [12]. It has been also associated with improved access, shorter visiting time, and higher patient satisfaction [4]. Furthermore, it has also been shown that primary care physicians who access telemedicine services reported higher confidence in the care of HIV-infected individuals and improved management of cases [13, 14]. Overall, research has established that there are high levels of patient satisfaction associated with telemedicine as a result of the reduction in travel, waiting time, and easy accessibility of specialists [15]. Recently, a retrospective review of outcomes of 234 referrals from the NHS network made to a multidisciplinary pediatric virtual clinic in the UK for complex case management as a model of care found the virtual support useful in complex treatment decision making in pediatric HIV infection [16]. In a systematic review of reviews that included 80 heterogeneous systematic reviews that evaluated the effectiveness of telemedicine, 21 reviews concluded that telemedicine is effective, 18 found that evidence is promising but incomplete, and others found that evidence is limited and inconsistent [17]. The authors of a Cochrane Collaborative research in 2015 concluded that the effectiveness of TM may depend on a number of different factors, including those related to the study population, the function of the intervention, and the healthcare provider and healthcare system involved in delivering the intervention [18]. We did not assess all the above-mentioned outcomes in this study. Furthermore, the individual case outcome was not linked to the attendance in the videoconference session; rather, we used the outcomes in the overall population, more at an ecologic level rather than an individual level. These are the potential limitations of the study.

Nonetheless, the study provided useful information to understand the role of telemedicine in providing specialized HIV care to children living with HIV/AIDS. It will be important to start such telemedicine initiatives in other states, so that CLHAs in rural parts of the country can avail of expert HIV care. The present study was a part of a larger study to assess the effectiveness of telemedicine in Maharashtra. Though it has been reported that patient acceptance, provider acceptance, and personnel training are barriers to telemedicine, we found that it was acceptable to healthcare providers and caregivers and cost-effective [19–21].

## 5. Conclusion

Our study clearly shows that the centers linked through telemedicine performed better in terms of patient care and treatment, with a lesser loss to follow-up and lesser deaths in CLHA. Overall, this pilot project of telemedicine for pediatric HIV has been proven to be acceptable, feasible, and effective in improving the quality of care for children living with HIV across the state of Maharashtra.

## Figures and Tables

**Table 1:** Description of the sample size of 31 regular linked and 28 nonlinked ART centers.

Nature	Total number of HIV cases ever registered^a^	Pediatric HIV cases ever registered^b^	Total number of HIV cases (October 2013-August 2015)	Pediatric HIV cases (October 2013–August 2015)^c^
Linked (31)	191,644	15,881 (8.3%)	36,100	2803 (7.8%)
Nonlinked (28)	197,320	15,985 (8.1%)	36,394	2608 (7.2%)

^a^These include HIV cases that were ever registered from the start of the center till August 2015. ^b^Proportion: total number of pediatric HIV cases ever registered/total number of HIV cases ever registered. ^c^Proportion: total number of pediatric HIV cases (October 2013 to August 2015)/total number of HIV cases registered (October 2013 to August 2015).

**Table 2:** Information about the population and other characteristics of 59 ART centers, Maharashtra, India.

	All	Nonlinked	Linked (irregular)	Linked (regular)	*p* value
*Information about children in these centers*					
Total	5411 (100)	2608 (100)	1365 (100)	1438 (100)	
Age groups					
0-4.9	937 (17)	430 (16)	249 (18)	258 (18)	0.49
5-9.9	1244 (23)	595 (23)	315 (23)	334 (23)	
10-14.9	1750 (32)	847 (32)	425 (31)	478 (33)	
15-18	1480 (27)	736 (28)	376 (28)	368 (26)	
Sex					
Male	2977 (55)	1386 (53)	795 (58)	796 (55)	0.009
Female	2434 (45)	1222 (47)	570 (42)	642 (45)	
*Information about centers*					
All	59	28	12	19	
Type of centers					
Civil hospital	25 (42)	3 (11)	7 (58)	15 (79)	<0.001
Medical college	17 (29)	14 (50)	3 (25)	0 (0)	
Subdistrict hospital	12 (20)	6 (21)	2 (17)	4 (21)	
Municipal corporation hospital	5 (8)	5 (18)	0 (0)	0 (0)	
Number of medical officers					
Median (range)	2 (1-3)	3 (1-3)	2 (1-3)	2 (1-3)	0.15
Education of the medical officer^∗^					
Postgraduate	52 (88)	23 (82)	10 (83)	19 (100)	0.13
Undergraduate	7 (12)	5 (18)	2 (17)	0 (0)	
Pediatrician in the ART center					
Yes	16 (27)	8 (29)	4 (33)	4 (21)	0.80
No	43 (73)	20 (71)	8 (67)	15 (79)	
Years of experience^∗∗^					
Median (range)	4 (0.2–10)	5 (0.3–10)	3 (0.4–8)	4 (0.2–9)	0.44
*Clinical features of the study population*					
Clinical group					
Pre-ART	1735 (32)	855 (33)	428 (31)	452 (31)	0.55
ART	3676 (68)	1753 (67)	937 (69)	986 (69)	
Baseline CD4 missing					
Yes	248 (5)	107 (4)	83 (6)	58 (4)	0.009
No	5163 (95)	2501 (96)	1282 (94)	1380 (96)	
Baseline CD4					
Median (IQR)	408 (209-765)	415 (213-765)	386 (179-748)	420 (217-771)	0.009
Latest CD4 missing					
Yes	1035 (19)	554 (21)	352 (26)	129 (9)	<0.001
No	4376 (81)	2054 (79)	1013 (74)	1309 (91)	
Latest CD4 count					
Median (IQR)	486 (267-801)	493 (268-834)	449.5 (242-791)	497.5 (295-776)	0.01

^∗^Highest education among all the medical officers; ^∗∗^highest years of experience among all the medical officers.

**Table 3:** Table showing certain quality of care indicators according to demographics in 1735 children in the pre-ART group in 59 ART centers, Maharashtra.

	*N*	Eligible but not initiated on ART			Baseline CD4 missing			Latest CD4 missing		
		Nonlinked	Linked (irregular)	Linked (regular)	Nonlinked	Linked (irregular)	Linked (regular)	Nonlinked	Linked (irregular)	Linked (regular)
*N*	1735	855	428	452	855	428	452	855	428	452
All		31 (4)	3 (1)	16 (4)^∗∗^	62 (7)	45 (11)	28 (6)^∗^	255 (30)	133 (31)	56 (12)^∗∗^
Age (years)										
0-4.9	244	4/113 (4)	1/69 (1)	1/62 (2)	9/113 (8)	12/69 (17)	4/62 (6)	33/113 (29)	23/69 (28)	14/62 (23)
5-9.9	581	7/290 (2)	1/137 (1)	2/154 (1)	16/290 (6)	7/137 (5)	9/154 (6)	85/290 (29)	43/137 (24)	16/154 (10)^∗∗^
10-14.9	582	13/288 (5)	1/141 (1)	10/153 (7)^∗^	19/288 (7)	14/141 (10)	10/153 (7)	86/288 (30)	44/141 (31)	16/153 (10)^∗∗^
15-18	328	7/164 (4)	0/81 (0)	3/83 (4)	18/164 (11)	12/81 (15)	5/83 (6)	51/164 (31)	23/81 (28)	10/83 (12)^∗^
Sex										
Male	965	20/481 (4)	3/242 (1)	6/242 (2)	34/481 (7)	30/242 (12)	20/242 (8)	128/481 (27)	83/242 (34)	33/242 (14)^∗∗^
Female	770	11/374 (3)	0/186 (0)	10/210 (5)^∗∗^	28/374 (7)	15/186 (8)	8/210 (4)	127/374 (34)	50/186 (27)	23/210 (11)^∗∗^
Nature of centers										
Civil hospital	659	0/65 (0)	2/197 (1)	14/395 (4)	13/65 (20)	15/199 (8)	24/395 (6)^∗∗^	22/65 (34)	59/199 (30)	50/395 (13)^∗∗^
Medical college	681	26/534 (5)	1/147 (1)^∗^	--	34/534 (6)	26/147 (18)^∗∗^	--	199/534 (37)	60/147 (41)	--
Subdistrict hospital	271	4/132 (3)	0/82 (0)	2/57 (4)	9/132 (7)	4/82 (5)	4/57 (7)	26/132 (20)	14/82 (17)	6/57 (11)
Municipal corporation hospital	124	1/124 (1)	--	--	6/124	--	--	8/124 (6)	--	--
Education										
Postgraduate	1465	25/672 (4)	3/341 (1)	16/452 (4)^∗^	48/672 (7)	20/341 (6)	28/452 (6)	214/672 (32)	104/341 (31)	56/452 (12)^∗∗^
Undergraduate	270	6/183 (3)	0/87 (0)	--	14/183 (8)	25/87 (29)^∗∗^	--	41/183 (22)	29/87 (33)	--
Pediatrician										
Yes	607	14/306 (5)	3/173 (2)	3/125 (2)	27/306 (9)	9/173 (5)	13/128 (10)	103/306 (34)	77/173 (45)	9/128 (7)^∗∗^
No	1128	17/549 (3)	0/255 (0)	13/311 (4)^∗∗^	35/549 (6)	36/255 (14)	15/324 (5)^∗∗^	152/549 (28)	56/255 (22)	47/324 (15)^∗∗^

^∗^ *p* < 0.05, ^∗∗^*p* < 0.01.

**Table 4:** Table showing the distribution of certain outcomes according to the demographics and characteristics of centers in 1735 children in the pre-ART group, Maharashtra.

	*N*	Alive			Dead			Lost to follow-up		
		Nonlinked	Linked (irregular)	Linked (regular)	Nonlinked	Linked (irregular)	Linked (regular)	Nonlinked	Linked (irregular)	Linked (regular)
*N*	1735	855	428	452	855	428	452	855	428	452
All		602 (70)	325 (76)	359 (79)^∗∗^	38 (4)	16 (4)	16 (4)	65 (8)	20 (5)	25 (6)
Age (years)										
0-4.9	244	75/113 (67)	45/69 (65)	47/62 (76)	4/113 (4)	4/69 (6)	1/62 (2)	8/113 (7)	3/69 (4)	2/62 (3)
5-9.9	581	212/290 (73)	115/137 (84)	132/154 (86)^∗∗^	10/290 (3)	3/137 (2)	4/154 (3)	20/290 (7)	5/137 (4)	8/154 (5)
10-14.9	582	210/288 (73)	111/141 (79)	119/153 (78)	15/288 (5)	3/141 (2)	5/153 (3)	20/288 (7)	8/141 (6)	9/153 (6)
15-18	328	105/164 (64)	54/81 (67)	61/83 (73)	9/164 (5)	6/81 (7)	6/83 (7)	17/164 (10)	4/81 (5)	6/83 (7)
Sex										
Male	965	343/481 (71)	178/242 (74)	188/242 (78)	22/481 (5)	10/242 (4)	9/242 (4)	37/481 (8)	7/242 (3)	18/242 (7)^∗^
Female	770	259/374 (69)	147/186 (79)	171/210 (81)^∗∗^	16/374 (4)	6/186 (3)	7/210 (3)	28/374 (7)	13/186 (7)	7/210 (3)
Type of centers										
Civil hospital	659	54/65 (83)	155/199 (78)	311/395 (79)	0/65 (0)	9/199 (5)	16/395 (4)	0/65 (0)	14/199 (7)	23/395 (6)
Medical college	681	371/534 (69)	96/147 (65)	--	26/534 (5)	7/147 (5)	--	44/534 (8)	3/144 (2)^∗∗^	--
Subdistrict hospital	271	111/132 (84)	74/82 (90)	48/57 (84)	5/132 (4)	0/82 (0)	0/57 (0)	5/132 (4)	3/82 (4)	2/57 (4)
Municipal corporation hospital	124	66/124 (53)	--	--	7/124 (6)	--	--	16/124 (13)	--	--
Education										
Postgraduate	1465	454/672 (68)	260/341 (76)	359/452 (79)^∗∗^	34/672 (5)	14/341 (4)	16/452 (4)	53/672 (8)	20/341 (6)	25/452 (6)
Undergraduate	270	148/183 (81)	65/87 (75)	--	4/183 (2)	2/87 (2)	--	12/183 (7)	0/87 (0)	--
Pediatrician										
Yes	607	215/306 (70)	132/173 (76)	105/128 (82)^∗^	21/306 (7)	11/173 (6)	6/128 (5)	14/306 (5)	10/173 (6)	5/128 (4)
No	1128	387/549 (70)	193/255 (76)	254/324 (78)^∗^	17/549 (3)	5/255 (2)	10/324 (3)	51/549 (9)	10/255 (4)	20/324 (6)^∗^

^∗^ *p* < 0.05, ^∗∗^*p* < 0.01.

**Table 5:** Table showing certain outcomes according to demographics in 3676 children on ART in 59 ART centers, Maharashtra.

	*N*	Alive			Dead			Lost to follow-up		
		Nonlinked	Linked (irregular)	Linked (regular)	Nonlinked	Linked (irregular)	Linked (regular)	Nonlinked	Linked (irregular)	Linked (regular)
*N*	3676	1753	937	986	1753	937	986	1753	937	986
All		1375 (78)	648 (69)	764 (77)^∗∗^	62 (4)	40 (4)	58 (6)^∗^	65 (4)	85 (9)	40 (4)^∗∗^
Age (years)										
0-4.9	693	236/317 (74)	115/180 (64)	150/196 (77)^∗^	12/317 (4)	12/180 (7)	9/196 (5)	15/317 (5)	16/180 (9)	9/196 (5)
5-9.9	663	245/305 (80)	125/178 (70)	133/180 (74)^∗^	8/305 (3)	4/178 (2)	15/180 (8)^∗∗^	8/305 (3)	12/178 (7)	6/180 (3)
10-14.9	1168	444/559 (79)	204/284 (72)	256/325 (79)^∗^	27/559 (5)	14/284 (5)	20/325 (6)	16/559 (3)	27/284 (10)	14/325 (4)^∗∗^
15-18	1152	450/572 (79)	204/295 (69)	225/285 (79)^∗∗^	15/572 (3)	10/295 (3)	14/285 (5)	26/572 (5)	30/295 (10)	11/285 (4)^∗∗^
Sex										
Male	2012	718/905 (79)	377/553 (68)	414/554 (75)^∗∗^	37/905 (4)	24/553 (4)	34/554 (6)	36/905 (4)	55/553 (10)	25/554 (5)^∗∗^
Female	1664	657/848 (77)	271/384 (71)	350/432 (81)^∗∗^	25/848 (3)	16/384 (4)	24/432(6)	29/848 (3)	30/384 (8)	15/432 (3)^∗∗^
Type of centers										
Civil hospital	1277	86/104 (83)	244 (74)	648/844 (77)	2/104 (2)	20/329 (6)	54/844 (6)	9/104 (9)	26/329 (8)	35/844 (4)^∗^
Medical college	1512	814/1019 (80)	302/493 (61)^∗∗^	--	28/1019 (3)	18/493 (4)	--	30/1019 (3)	55/493 (11)^∗∗^	--
Subdistrict hospital	492	196/235 (83)	102/115 (89)	116/142 (82)	18/235 (8)	2/115 (2)	4/142 (3)	8/235 (3)	4/115 (3)	5/142 (4)
Municipal corporation hospital	395	279/395 (71)	--	--	14/395 (4)	--	--	18/377 (5)	--	--
Education										
Postgraduate	2974	1053/1375 (77)	481/613 (78)	764/986 (77)	46/1375 (3)	28/613 (5)	58/928 (6)^∗^	50/1375 (4)	31/613 (5)	40/986 (4)
Undergraduate	702	322/378 (85)	167/324 (52)^∗∗^	--	16/378 (4)	12/312 (4)	--	15/378 (4)	54/324 (17)^∗∗^	--
Pediatrician										
Yes	1248	558/703 (79)	233/293 (80)	204/252 (81)	22/703 (3)	16/293 (5)	13/252 (5)	19/703 (3)	11/293 (4)	6/252 (2)
No	2428	817/1050 (78)	415/644 (64)	560/734 (76)^∗∗^	40/1050 (4)	24/644 (4)	45/734 (6)^∗^	46/1050 (4)	74/644 (11)	34/734 (5)^∗∗^

^∗^ *p* < 0.05, ^∗∗^*p* < 0.01.

**Table 6:** Table showing certain quality of care indicators according to demographics in 3676 children on ART in 59 ART centers, Maharashtra.

	*N*	Baseline CD4 missing			Latest CD4 missing		
		Nonlinked	Linked (irregular)	Linked (regular)	Nonlinked	Linked (irregular)	Linked (regular)
*N*	3676	1753	937	986	1753	937	986
All		45 (3)	38 (4)	30 (3)	299 (17)	219 (23)	73 (7)^∗∗^
Age (years)							
0-4.9	693	18/317 (6)	6/180 (3)	12/196 (6)	68/317 (21)	56/180 (31)	23/196 (12)^∗∗^
5-9.9	663	7/305 (2)	8/178 (4)	4/180 (2)	62/305 (20)	51/178 (29)	16/180 (9)^∗∗^
10-14.9	1168	9/559 (2)	14/284 (5)	11/325 (3)^∗^	82/559 (15)	55/284 (19)	17/325 (5)^∗∗^
15-18	1152	11/572 (2)	10/295 (3)	3/285 (1)	87/572 (15)	57/295 (19)	17/285 (6)^∗∗^
Sex							
Male	2012	24/905 (3)	24/553 (4)	16/554 (3)	159/905 (18)	138/553 (25)	40/554 (7)^∗∗^
Female	1664	21/848 (2)	14/384 (4)	14/432 (3)	140/848 (17)	81/384 (21)	33/432 (8)^∗∗^
Type of centers							
Civil hospital	1277	13/104 (13)	4/329 (1)	26/844 (3)^∗∗^	19/104 (18)	60/329 (18)	47/844 (6)^∗∗^
Medical college	1512	16/1019 (2)	34/493 (7)^∗∗^	--	240/1019 (24)	159/493 (32)^∗∗^	--
Subdistrict hospital	492	10/235 (4)	0/115 (0)	4/138 (3)	34/235 (14)	0/115 (0)	26/142 (18)^∗∗^
Municipal corporation hospital	395	6/395 (2)	--	--	6/395 (2)	--	--
Education							
Postgraduate	2974	27/1375 (2)	4/61 (1)	30/986 (3)^∗∗^	209/1375 (15)	135/613 (22)	73/986 (7)^∗∗^
Undergraduate	702	18/378 (5)	34/324 (10)^∗∗^	--	90/378 (24)	84/324 (26)	--
Pediatrician							
Yes	1248	15/703 (2)	3/293 (1)	14/252 (6)^∗∗^	79/703 (11)	131/293 (45)	4/252 (2)^∗∗^
No	2428	30/1050 (3)	35/644 (5)	16/734 (2)^∗∗^	220/1050 (21)	88/644 (14)	69/734 (9)^∗∗^

^∗^ *p* < 0.05, ^∗∗^*p* < 0.01.

## Data Availability

Access to data is restricted since this included information from HIV-infected children; thus, patient privacy and legal issues are important.
